# Does the presence and mix of destinations influence walking and physical activity? 

**DOI:** 10.1186/s12966-015-0279-0

**Published:** 2015-09-17

**Authors:** Tania Louise King, Rebecca Jodie Bentley, Lukar Ezra Thornton, Anne Marie Kavanagh

**Affiliations:** Gender and Women’s Health, Centre for Health and Society, Academic Centre for Health Inequity, Melbourne School of Population Health, University of Melbourne, Melbourne, Australia; Centre for Physical Activity and Nutrition Research, School of Exercise and Nutrition Sciences, Deakin University, Burwood, VIC Australia

**Keywords:** Walking, Physical activity, Geographic information systems, Multilevel analysis, Built environment, Destinations

## Abstract

**Background:**

Local destinations have previously been shown to be associated with higher levels of both physical activity and walking, but little is known about how specific destinations are related to activity. This study examined associations between types and mix of destinations and both walking frequency and physical activity.

**Method:**

The sample consisted of 2349 residents of 50 urban areas in metropolitan Melbourne, Australia. Using geographic information systems, seven types of destinations were examined within three network buffers (400 meters (m), 800 m and 1200 m) of respondents’ homes. Multilevel logistic regression was used to estimate effects of each destination type separately, as well as destination mix (variety) on: 1) likelihood of walking for at least 10 min ≥ 4/week; 2) likelihood of being sufficiently physically active. All models were adjusted for potential confounders.

**Results:**

All destination types were positively associated with walking frequency, and physical activity sufficiency at 1200 m. For the 800 m buffer: all destinations except transport stops and sports facilities were significantly associated with physical activity, while all except sports facilities were associated with walking frequency; at 400 m, café/takeaway food stores and transport stops were associated with walking frequency and physical activity sufficiency, and sports facilities were also associated with walking frequency. Strongest associations for both outcomes were observed for community resources and small food stores at both 800 m and 1200 m. For all buffer distances: greater mix was associated with greater walking frequency. Inclusion of walking in physical activity models led to attenuation of associations.

**Conclusions:**

The results of this analysis indicate that there is an association between destinations and both walking frequency and physical activity sufficiency, and that this relationship varies by destination type. It is also clear that greater mix of destinations positively predicts walking frequency and physical activity sufficiency.

**Electronic supplementary material:**

The online version of this article (doi:10.1186/s12966-015-0279-0) contains supplementary material, which is available to authorized users.

## Background

Physical inactivity is known to be associated with a range of serious health risks and diseases [1]; indeed it has been claimed that together with smoking, physical inactivity may be the most significant, modifiable determinant of all-cause mortality and chronic morbidity [1, 2]. Walking is the most common form of physical activity in both Australia [3–5] and elsewhere such as the United States [1, 6, 7], and is known to offer many potential health benefits [8–10].

The local neighbourhood is an important setting for physical activity, and there is mounting evidence that elements of the built environment are associated with walking and physical activity [11–15]. Destinations are an increasing focus of investigation: if destinations can encourage more active travel, such as walking in neighbourhoods, it is possible that residents may be more likely to meet their physical activity needs. Increased accessibility to destinations is known to have positive associations with walking behaviours [16–20]. These associations between destinations, and walking, have been reported in many countries including Australia [18, 21, 22], Belgium [23], Japan [24], the US [25–28], New Zealand [29] and the United Kingdom [30] on a number of indicators including: the presence of destinations within walking distance of home [18, 27, 31], proximity to destinations [26, 32, 33], and density of destinations [27].

Other researchers have observed that walking is more likely to be influenced by the type and mix of destinations than simply the presence of destinations [21]. Most previous studies of destination mix (often referred to as *number of types of businesses/destinations*) indicate that increased destination mix is associated with increased walking [18, 32, 34, 35], however there are some contrary results, with destination mix having no effect on walking levels among older Australians (aged 65–84 years) [19].

There are some important limitations with the current evidence-base that this study seeks to address. First, it is unclear exactly what types of destinations produce the greatest effects on physical activity and walking, as few previous studies have looked at multiple, specific destinations. Other researchers have observed that most studies have focused on ‘commercial destinations’ such as shops and services [36], or destinations that are intuitively associated with walking [37]. Non-commercial destinations such as community resources (*e.g.,* libraries) may also influence neighbourhood physical activity and walking in adults. Second, it is difficult to judge the distance that residents might be prepared to walk to access destinations as most previous studies have used just one, sometimes two catchments/buffer distances. A recent review highlighted the need for research into potential threshold distances at which destinations might encourage walking [38]. Thirdly, there is a need for more sophisticated methods of measuring destination mix. While some authors have examined the mix of destinations, they have typically relied on relatively simple measures of the number of unique types of destinations within a specific distance of respondents’ homes [18, 37]. However, some destinations such as transport stops may be more common than others (*e.g.,* supermarkets). Mix may be better captured by a measure that accounts for the relative frequency of the different types of destinations. We are aware of only one study that has considered how access to multiple destinations of a particular type might influence walking more than access to only one [29], however this incorporated mix into a broader index of destination accessibility.

To address some of the identified gaps in previous methods this study sought to:

1. Identify which destinations (of supermarkets, small food stores, transport stops and stations, community resources, cafes and takeaway food stores, sporting facilities, and educational facilities) within residential neighbourhoods are associated with walking for 10 min or more, at least four times a weeks.
2. Assess the extent to which the hypothesized associations between destinations and walking frequency translate into associations between destinations and physical activity sufficiency (given that walking is the most common form of physical activity).
3. Understand how the mix of destinations is associated with walking frequency and physical activity, where mix takes into account the relative frequency of different destination types across the sample.

## Methods

### Datasets

#### Individual-level data

Individual-level variables from the Victorian Lifestyle and Neighbourhood Environment Study (VicLANES) dataset were used. The methods used in the VicLANES study and details of the sample have been documented previously [39–42].

In brief, VicLANES was a large, multilevel study conducted in 2003–2004 across the 21 innermost local government areas (LGAs) in Melbourne, Australia. Fifty census collection districts (known as CCDs, at the time of the study these were the smallest geographic unit of measurement used by the Australian Bureau of Statistics (ABS)) were randomly selected from the sample of LGAs stratified by a household measure of low income (<$400/week). Surveys about physical activity were sent to 4005 residents 18 years and over, who were randomly selected from the electoral roll (voting is compulsory for all Australians over 18 years, and it is estimated that 97.7 % of those eligible to vote are enrolled) [43]. A 58.7 % valid completion rate was achieved, with 2349 residents returning a completed survey.

### Destination data

Destination information came from the VicLANES environmental audit, and publicly available spatial datasets such as Ausway™ and PSMA. The VicLANES environmental audit has been reported previously [41], and involved a team of trained auditors collecting detailed information on different food shops selling food for consumption within the home.

The destination variables included in the dataset were classified into seven categories: educational facilities, café/takeaway stores, transport stops, supermarkets, sports facilities, community resources, small food stores. Data for supermarkets, small food stores and café/takeaway stores came from VicLANES. Destinations included in the education, community resource and sport layers came from Ausway™, producers of Australian street directories. Transport data came from Metlink (the public transport operator for the Victorian State Government), and PSMA (Public Service Mapping Authority). A summary of these variables is contained in Additional file 1: Table S1.

The *community resources* category included the following: maternal and child health centres; community health centres; community centres; post offices; places of worship; cinemas, theatres and art galleries; public libraries. Based on advice from Ausway™ that ‘core’ community services such as schools, post offices, community services were complete, but non-core services such as restaurants were not, we chose ‘*community resource’* features that were deemed both ‘core’ and a potential destination for walking.

The *education* category was comprised of the following Ausway™ derived points of interest: schools (primary and secondary); childcare and kindergartens; other places of education such as universities and TAFE^1^ campuses. *Public transport* contained tram and bus stops and railway stations. A *supermarket* was defined as a food store selling fresh produce with four or more checkouts. The *small food store* category included: fruit and vegetable shops; butchers and fishmongers; bakeries; small grocery stores (less than 4 checkouts); convenience store (corner store, fuel station with convenience food); specialty shops (such as delicatessens, health food stores, ethnic food stores). *Café and restaurant/takeaway* excluded restaurants that were only dine-in premises. The *sports category* contained information on all swimming pools and tennis courts in the study areas.

### Outcome measures

#### Walking frequency

A closed response question asked respondents about their frequency of walking for at least 10 min in the previous month. Respondents were required to tick one of six response categories: never, about once or twice, about once a week, about 2–3 times a week, about 4–5 times a week, every day. Responses were dichotomized to ‘less than four times a week’ (≤4/week) and ‘four times a week or more’ (≥4/week). The cut-off response category (4–5 times a week) for this dichotomization closely approximates the number of sessions (at least five) recommended to meet physical activity sufficiency [1, 44].

#### Physical activity sufficiency

Using items from the Active Australia Survey, respondents were asked to indicate the frequency and duration of their participation in walking, vigorous physical activity, moderate physical activity, vigorous garden or yard work. These items were then used to produce a measure of overall physical activity sufficiency. The Active Australia Questionnaire has been used in national surveys, and demonstrates very good reliability and validity [44].

Australian and international guidelines recommend that a person participate in at least 30 min of moderate to vigorous intensity activity most days of the week, for a total of at least 150 min of activity [1, 44, 45]. According to the Active Australia Survey guidelines, physical activity sufficiency for health can be measured in two ways [44]: 1) measured as total time engaged in physical activity (at least 150 min for sufficiency); 2) measured as the total time across the total number of sessions (at least 150 min across at least five sessions). We have chosen to use the combined measure of time and number of sessions (at least 150 min of at least moderate intensity activity across at least five session week) [46, 47], because it matches guidelines for physical activity sufficiency.

In accordance with the Active Australia Survey administration and implementation guidelines, VicLANES responses were converted to total amount of time (minutes) engaged in each activity, and summed, with vigorous activity weighted by a factor of two [4, 44]. Respondents were then categorized in one of two categories: those reporting less than 150 min of at least moderate activity were classified as ‘insufficiently active’; those with at least 150 min of at least moderate activity across at least five sessions were classified as ‘sufficiently physically active’.

### Spatial analysis: buffer generation and network analysis

Geographic information systems (GIS) software was used to generate network buffers at 400 m, 800 m, 1200 m distances around each individual’s residence. Network analysis was then conducted to identify the number of destinations within each distance of respondents’ homes. Destination counts were then extracted from GIS, and formed the exposure variables, described henceforth.

### Exposure variables: destinations and destination mix

The destination counts arising from the spatial analysis were positively skewed and were therefore modelled as ordinal variables. In the first instance we sought to model the exposure variables as tertiles, as tertiles enable exploration of a dose response gradient. Due to the way that responses clustered around certain values, the use of tertiles was not always possible (*e.g.,* where responses were highly dominated by 0). In such instances variables were modelled as binary exposures (refer to Table 1 for list of exposure variable types and cut points).

**Table 1:** Summary of exposure variables

Exposure variable	Buffer (m)	Mean (SD)	Range	Median (IQR)	Variable type (modelled as):	Category cut points		
Education	400	0.59 (0.97)	0–7	0 (0, 1)	Binary	0	1+	
Education	800	2.31 (2.31)	0–14	2 (1, 3)	Tertile	0–1	2–3	4+
Education	1200	5.05 (3.77)	0–20	4 (2, 7)	Tertile	0–3	4–6	7+
Café/takeaway stores	400	0.71 (1.59)	0–16	0 (0, 1)	Binary	0	1+	
Café/takeaway stores	800	4.69 (8.55)	0–56	1 (0, 5)	Tertile	0	1–3	4+
Café/takeaway stores	1200	12.14 (15.66)	0–73	7 (1, 16)	Tertile	0–3	4–11	12+
Transport stops	400	3.5 (3.13)	0–18	3 (1, 6)	Tertile	0	1–3	4+
Transport stops	800	13.42 (8.09)	0–45	13 (8, 18)	Tertile	0–10	11–16	17+
Transport stops	1200	29.75 (15.78)	0–81	27 (19, 39)	Tertile	0–23	24–35	36+
Supermarkets	400	0.05 (0.29)	0–2	0 (0, 0)	Binary	0	1+	
Supermarkets	800	0.31 (0.67)	0–4	0 (0, 0)	Binary	0	1+	
Supermarkets	1200	0.80 (1.02)	0–4	0 (0, 1)	Binary	0	1+	
Sports facilities	400	0.20 (0.52)	0–4	0 (0, 0)	Binary	0	1+	
Sports facilities	800	0.78 (1.05)	0–5	0 (0, 1)	Binary	0	1+	
Sports facilities	1200	1.72 (1.56)	0–8	2 (0, 3)	Tertile	0	1–2	3+
Community resources	400	0.51 (1.01)	0–5	0 (0, 1)	Binary	0	1+	
Community resources	800	2.54 (2.97)	0–15	2 (0, 4)	Tertile	0–1	2–3	4+
Community resources	1200	5.99 (5.56)	0–31	5 (1, 9)	Tertile	0–2	3–7	8+
Small food stores	400	0.71 (1.70)	0–17	0 (0, 1)	Binary	0	1+	
Small food stores	800	4.23 (6.13)	0–55	2 (0, 5)	Tertile	0	1–2	3+
Small food stores	1200	10.48 (11.28)	0–58	7 (2, 15)	Tertile	0–3	4–11	12+

### Exposure variables: derivation of mix measure

This study used a measure of destination mix that accounted for variation in the frequency of different destination types. The chief reason for measuring mix in this way was to equalize the effect of different destination types, particularly those more widespread than others. Some destinations (*i.e.,* transport destinations) were abundant, whereas others (*i.e.,* supermarkets) were sparse. The mix measure was constructed by:

1. Calculating the median number of destinations for each type at each buffer distance
2. For each person, assigning a value of 1 when the number of destinations were above the median and 0 if equal or below the median for each type
3. Summing each of the values derived in point (2), to create a mix variable with a range of 0–7

### Confounders

Based on the literature, several covariates were included in the models as potential confounders because they are likely to be related to walking frequency, physical activity and destination distribution. These were: age (18–24 years, 25–34 years, 35–44 years, 45–54 years, 55–64 years, 64 years and over); sex; country of birth (born in Australia, born in a country other than Australia); education (bachelor degree or higher, diploma, vocational training, and no post school qualification); household type (single adult-no children, single adult with children, two or more adults-no children, two or more adults with children); disability/injury that prevents exercise (yes, no); area disadvantage (least disadvantaged, mid disadvantaged and most disadvantaged); and dominant household occupation (professional, white-collar employee, blue-collar employee, not in labour force). The ‘not in labour force’ category included retirees, students, unemployed, those not looking for, or unable to work.

### Statistical analysis

All statistical analyses were conducted in Stata IC 10.0. Pregnant women (n = 22) were excluded because their walking and physical activity levels may have been altered by their pregnancy status. One CCD from just outside the central business district (CBD) of Melbourne was omitted from the final analysis (n = 14) as this CCD’s catchment area encapsulated almost the entire CBD, and the number of features/destinations contained in its catchment was irregularly high. Missing data for the other variables ranged from 0.5–2.9 %, with the exception of the disability item, for which missing data amounted to 6.1 %. Eight respondents for whom there was no walking data were excluded, resulting in an analytical sample of 2305 respondents, and 49 CCDs for the walking analysis. For the physical activity analysis, data was missing from 373 respondents (15.9 %), resulting in an analytical sample of 1976.

Descriptive analyses included cross-tabulations between the outcomes and individual covariates and destination exposure variables. Using the xtmelogit commands in Stata, multilevel logistic regression was conducted to examine associations between the outcomes and exposure variables. All models adjusted for the above mentioned confounders and area level clustering. Odds ratios and 95 % confidence intervals are reported for the effect estimates. The referent category for the walking outcome was the lowest walking category (walking < 4/month); for the physical activity variable the referent was ‘insufficient physical activity’, while the referent categories for the exposure variables were the lowest category (‘Tertile 1’ for tertiles/‘0’ for binary categories). Destination mix was modelled as a continuous variable.

## Results

### Summary of destination variables

Cafes/takeaway stores, transport stops and small food stores were the most common destinations, while supermarkets and sports facilities were least common (see Table 1).

### Individual covariates and destinations

Refer to Additional file 2: Table S2 for a summary of covariates by destination types. Broadly, neighbourhoods with most destinations were characterized by: high area disadvantage; adults living alone, those aged 65 years or more; highly educated professionals.

### Individual covariates and destination mix

Table 2 presents a summary of covariates by destination mix. The associations were similar to those found for the individual destinations. Neighbourhoods with the greatest mix of destinations were more likely to be areas of high disadvantage, and contain a higher proportion of people: aged 65 years or more; living alone; with a bachelor degree or higher.

**Table 2:** Covariates by destination mix exposure variable

Covariates		Total	Mix 400 m	Mix 800 m	Mix 1200 m
		N (%)	mean (SD)	mean (SD)	mean (SD)
Sex	Male	1015 (44.0)	1.9 (1.8)	2.8 (2.3)	3.1 (2.4)
	Female	1290 (56.0)	1.9 (1.8)	2.7 (2.2)	3.1 (2.4)
Age-group	18–24 years	182 (7.9)	1.6 (1.6)	2.3 (2.1)	2.6 (2.3)
	25–34 years	395 (17.1)	2.0 (1.7)	2.9 (2.2)	3.4 (2.3)
	35–44 years	492 (21.3)	2.0 (1.7)	3.0 (2.2)	3.3 (2.3)
	45–54 years	495 (21.5)	1.8 (1.7)	2.5 (2.2)	2.9 (2.4)
	55–64 years	391 (17.0)	1.8 (1.7)	2.5 (2.3)	2.8 (2.5)
	65+ years	350 (15.2)	2.2 (2.0)	3.2 (2.3)	3.5 (2.4)
Country of birth	Elsewhere	663 (28.9)	1.9 (1.7)	2.6 (2.2)	3.0 (2.4)
	Australia	1631 (71.1)	1.9 (1.8)	2.8 (2.3)	3.2 (2.4)
Education	Bachelor degree +	719 (32.1)	1.9 (1.8)	3.1 (2.3)	3.6 (2.4)
	Diploma	257 (11.5)	1.8 (1.8)	2.8 (2.3)	3.2 (2.4)
	Vocational	431 (19.3)	1.9 (1.8)	2.5 (2.2)	2.8 (2.3)
	No post school qualifications	831 (37.1)	1.9 (1.7)	2.5 (2.2)	2.9 (2.3)
Dominant household occupation	Professionals	1060 (47.1)	1.9 (1.7)	2.9 (2.3)	3.3 (2.4)
	White-collar	352 (15.6)	1.9 (1.7)	2.5 (2.2)	2.9 (2.4)
	Blue-collar	243 (10.8)	1.7 (1.5)	2.3 (2.1)	2.5 (2.2)
	Not in labour force	597 (26.5)	2.1 (1.9)	2.9 (2.3)	3.1 (2.3)
Household type	Single adult, no children	397 (17.6)	2.6 (1.9)	3.8 (2.2)	4.1 (2.3)
	Single adult, children	133 (5.9)	1.8 (1.7)	2.3 (2.2)	2.8 (2.2)
	2+ adults, no children	947 (42.0)	1.8 (1.7)	2.6 (2.2)	3.0 (2.4)
	2+ adults, children	778 (34.5)	1.7 (1.7)	2.4 (2.2)	2.8 (2.4)
Injury/disability	No	1675 (77.4)	1.8 (1.7)	2.7 (2.3)	3.0 (2.4)
	Yes	489 (22.6)	2.2 (1.9)	2.9 (2.2)	3.4 (2.3)
Area disadvantage	Low	834 (36.2)	1.5 (1.7)	2.2 (2.1)	2.3 (2.4)
	Mid	772 (33.5)	1.9 (1.7)	2.6 (2.3)	3.4 (2.5)
	High	699 (30.3)	2.4 (1.9)	3.6 (2.1)	3.8 (2.0)
Total sample		2305 (100.0)	1.9 (1.8)	2.7 (2.3)	3.1 (2.4)

### Frequency of walking in areas characterized by destinations

For most destinations, a higher proportion of those walking ≥4/week and sufficiently active were in the higher exposure category (*e.g.,* tertile three). For example, for educational facilities within both the 800 m and 1200 m buffer, a higher proportion of those respondents both walking most frequently (≥4/week), and sufficiently physically active, were in the tertile or category with the most facilities (see Table 3).

**Table 3:** Summary statistics for outcome and exposures

	Exposure variable	Exposure category	Walking frequency			Physical activity sufficiency		
			Total	<4/week	≥4/week	Total	Insufficient	Sufficient
			N	%	%	N	%	%
400	Education	0 education facilities	1469	65.5	61.5	1266	64.1	66.8
		1 + education facilities	836	34.5	38.5	710	35.9	33.2
800	Education	Tertile 1	996	46.3	39.2	869	44.0	48.1
		Tertile 2	808	33.1	37.6	676	34.2	32.9
		Tertile 3	501	20.7	23.1	431	21.8	19.0
1200	Education	Tertile 1	939	44.7	35.5	815	41.2	46.5
		Tertile 2	698	27.7	33.6	593	30.0	27.8
		Tertile 3	668	27.6	30.8	568	28.7	25.7
400	Cafes/takeaway stores	0 cafes/takeaway	1659	75.5	67.4	1435	72.6	75.6
		1 + cafes/takeaway	646	24.5	32.6	541	27.4	24.5
800	Cafes/takeaway stores	Tertile 1	798	38.7	29.3	707	35.8	39.2
		Tertile 2	761	32.6	33.5	632	32.0	32.2
		Tertile 3	746	28.7	37.1	637	32.2	28.7
1200	Cafes/takeaway stores	Tertile 1	827	39.1	31.7	731	37.0	40.0
		Tertile 2	714	31.9	29.8	592	30.0	31.3
		Tertile 3	764	29.1	38.4	653	33.1	28.7
400	Transport stops/stations	Tertile 1	561	27.6	20.0	496	25.1	28.4
		Tertile 2	684	29.1	30.4	584	29.6	28.3
		Tertile 3	1060	43.3	49.6	896	45.3	43.3
800	Transport stops/stations	Tertile 1	893	41.0	35.7	784	39.7	41.4
		Tertile 2	730	32.5	30.6	630	31.9	31.9
		Tertile 3	682	26.5	33.6	562	28.4	26.8
1200	Transport stops/stations	Tertile 1	825	39.3	31.2	724	36.6	40.0
		Tertile 2	760	31.4	35.0	651	33.0	31.2
		Tertile 3	720	29.3	33.7	601	30.4	28.9
400	Supermarkets	0 supermarkets	2219	96.9	95.4	1905	96.4	96.4
		1 + supermarkets	86	3.1	4.6	71	3.6	3.6
800	Supermarkets	0 supermarkets	1812	82.2	74.0	1557	78.8	81.5
		1 + supermarkets	493	17.8	26.0	419	21.2	18.5
1200	Supermarkets	0 supermarkets	1218	56.3	48.4	1056	53.4	57.5
		1 + supermarkets	1087	43.7	51.7	920	46.6	42.5
400	Sport facilities	0 sport facilities	1938	86.1	81.5	1678	84.9	84.9
		1 + sport facilities	367	13.9	18.5	298	15.1	15.1
800	Sport facilities	0 sport facilities	1296	59.0	52.7	1136	57.5	59.2
		1 + sport facilities	1009	41.0	47.4	840	42.5	40.8
1200	Sport facilities	Tertile 1	639	31.7	22.5	563	28.5	32.5
		Tertile 2	1045	43.2	48.2	900	45.6	42.2
		Tertile 3	621	25.1	29.3	513	26.0	25.3
400	Community resources	0 comm. resources	1644	73.6	68.4	1427	72.2	74.7
		1 + comm. resources	661	26.4	31.6	549	27.8	25.3
800	Community resources	Tertile 1	733	37.3	24.6	654	33.1	38.6
		Tertile 2	692	28.0	32.7	593	30.0	27.7
		Tertile 3	880	34.8	42.6	729	36.9	33.7
1200	Community resources	Tertile 1	796	39.4	28.1	707	35.8	40.8
		Tertile 2	744	32.2	32.3	628	31.8	32.4
		Tertile 3	765	28.3	39.5	641	32.4	26.8
400	Small food stores	0 small food stores	1578	70.4	65.9	1370	69.3	69.9
		1 + small food stores	727	29.6	34.1	606	30.7	30.1
800	Small food stores	Tertile 1	575	28.7	20.0	520	26.3	29.5
		Tertile 2	758	33.5	32.1	636	32.2	33.0
		Tertile 3	972	37.8	47.9	820	41.5	37.5
1200	Small food stores	Tertile 1	814	39.8	29.4	726	36.7	40.5
		Tertile 2	761	32.2	34.0	625	31.6	32.8
		Tertile 3	730	28.0	36.5	625	31.6	26.7

### Multi-level logistic regression analysis: Specific types of destination

There was a clear gradient for each destination type: more destinations were associated with greater odds of walking ≥ 4/week, and greater odds of being sufficiently physically active (refer to Table 4).

**Table 4:** Multilevel logistic regression odds ratios for each destination, across three buffer distances^1^

			Odds ratios (CI)		
Exposure variable	Buffer distance	Response category	Walking four times a week or more^2^	Physical activity sufficiency^3^	Physical activity sufficiency adjusted for walking
Education	400	0 education facilities	1.00	1.00	1.00
		1+ education facilities	1.11 (0.90, 1.38), p = 0.326	1.16 (0.92, 1.46), p = 0.217	1.10 (0.85, 1.43), p = 0.470
Education	800	Tertile 1	1.00	1.00	1.00
		Tertile 2	1.40 (1.11, 1.77), p = 0.005	1.45 (1.14, 1.83), p = 0.002	1.28 (0.97, 1.70), p = 0.081
		Tertile 3	1.27 (0.96, 1.69), p = 0.099	1.50 (1.13, 1.99), p = 0.005	1.42 (1.03, 1.96), p = 0.035
Education	1200	Tertile 1	1.00	1.00	1.00
		Tertile 2	1.77 (1.39, 2.24), p = 0.000	1.60 (1.24, 2.06), p = 0.000	1.28 (0.95, 1.73), p = 0.111
		Tertile 3	1.54 (1.19, 1.98), p = 0.001	1.44 (1.10, 1.89), p = 0.009	1.25 (0.90, 1.72), 0.179
Cafes/takeaway stores	400	0 cafes/takeaway	1.00	1.00	1.00
		1+ cafes/takeaway	1.47 (1.18, 1.83), p = 0.001	1.36 (1.08, 1.73), p = 0.009	1.23 (0.94, 1.62), p = 0.130
Cafes/takeaway stores	800	Tertile 1	1.00	1.00	1.00
		Tertile 2	1.34 (1.04, 1.73), p = 0.022	1.35 (1.04, 1.75), p = 0.023	1.18 (0.88, 1.60), p = 0.274
		Tertile 3	1.80 (1.38, 2.34), p = 0.000	1.60 (1.22, 2.09), p = 0.001	1.28 (0.94, 1.75), p = 0.120
Cafes/takeaway stores	1200	Tertile 1	1.00	1.00	1.00
		Tertile 2	1.18 (0.92, 1.53), p = 0.198	1.07 (0.82, 1.39), p = 0.622	1.00 (0.74, 1.37), p = 0.985
		Tertile 3	1.66 (1.27, 2.17), p = 0.000	1.61 (1.23, 2.12), p = 0.001	1.37 (0.99, 1.88), p = 0.057
Transport stops/stations	400	Tertile 1	1.00	1.00	1.00
		Tertile 2	1.59 (1.22, 2.08), p = 0.001	1.56 (1.18, 2.06), p = 0.002	1.30 (0.94, 1.79), p = 0.111
		Tertile 3	1.58 (1.22, 2.06), p = 0.001	1.54 (1.17, 2.01), p = 0.002	1.32 (0.97, 1.81), p = 0.077
Transport stops/stations	800	Tertile 1	1.00	1.00	1.00
		Tertile 2	1.06 (0.83, 1.37), p = 0.628	1.09 (0.84, 1.40), p = 0.531	1.05 (0.78, 1.40), p = 0.748
		Tertile 3	1.35 (1.02, 1.79), p = 0.035	1.13 (0.84, 1.52), p = 0.409	1.02 (0.73, 1.42), p = 0.923
Transport stops/stations	1200	Tertile 1	1.00	1.00	1.00
		Tertile 2	1.47 (1.14, 1.89), p = 0.003	1.49 (1.15, 1.93), p = 0.003	1.29 (0.95, 1.75), p = 0.104
		Tertile 3	1.46 (1.11, 1.93), p = 0.007	1.33 (0.99, 1.79), p = 0.058	1.19 (0.85, 1.68), p = 0.309
Supermarkets	400	0 supermarkets	1.00	1.00	1.00
		1 + supermarkets	1.23 (0.73, 2.08), p = 0.441	0.82 (0.47, 1.44), p = 0.495	0.72 (0.38, 1.37), p = 0.317
Supermarkets	800	0 supermarkets	1.00	1.00	1.00
		1 + supermarkets	1.69 (1.30, 2.20), p = 0.000	1.43 (1.08, 1.88), p = 0.012	1.15 (0.83, 1.58), p = 0.406
Supermarkets	1200	0 supermarkets	1.00	1.00	1.00
		1 + supermarkets	1.40 (1.12, 1.77), p = 0.004	1.53 (1.22, 1.91), p = 0.000	1.41 (1.09, 1.83), p = 0.009
Sport facilities	400	0 sport facilities	1.00	1.00	1.00
		1 + sport facilities	1.33 (1.00, 1.76), p = 0.049	0.93 (0.68, 1.27), p = 0.647	0.76 (0.53, 1.09), p = 0.137
Sport facilities	800	0 sport facilities	1.00	1.00	1.00
		1 + sport facilities	1.21 (0.97, 1.52), p = 0.089	1.17 (0.93, 1.47), p = 0.178	1.05 (0.81, 1.36), p = 0.707
Sport facilities	1200	Tertile 1	1.00	1.00	1.00
		Tertile 2	1.53 (1.19, 1.98), p = 0.001	1.58 (1.22, 2.03), p = 0.000	1.39 (1.03, 1.87), p = 0.030
		Tertile 3	1.66 (1.22, 2.25), p = 0.001	1.53 (1.14, 2.06), p = 0.005	1.22 (0.86, 1.73), p = 0.270
Community resources	400	0 comm. resources	1.00	1.00	1.00
		1 + comm. resources	1.12 (0.89, 1.40), p = 0.334	1.06 (0.82, 1.36), p = 0.661	1.04 (0.78, 1.37), p = 0.800
Community resources	800	Tertile 1	1.00	1.00	1.00
		Tertile 2	1.85 (1.44, 2.37), p = 0.000	1.82 (1.40, 2.37), p = 0.000	1.55 (1.13, 2.12), p = 0.006
		Tertile 3	1.92 (1.49, 2.48), p = 0.000	1.58 (1.2, 2.07), p = 0.001	1.23 (0.88, 1.70), p = 0.222
Community resources	1200	Tertile 1	1.00	1.00	1.00
		Tertile 2	1.41 (1.11, 1.79), p = 0.006	1.45 (1.12, 1.89), p = 0.005	1.32 (0.96, 1.81), p = 0.084
		Tertile 3	2.20 (1.71, 2.83), p = 0.000	1.97 (1.49, 2.58), p = 0.000	1.47 (1.06, 2.04), p = 0.023
Small food stores	400	0 small food stores	1.00	1.00	1.00
		1 + small food stores	1.12 (0.9, 1.4), p = 0.296	1.07 (0.85, 1.35), p = 0.573	1.04 (0.8, 1.37), p = 0.747
Small food stores	800	Tertile 1	1.00	1.00	1.00
		Tertile 2	1.42 (1.08, 1.85), p = 0.011	1.39 (1.05, 1.85), p = 0.020	1.28 (0.92, 1.78), p = 0.146
		Tertile 3	1.85 (1.41, 2.44), p = 0.000	1.70 (1.29, 2.26), p = 0.000	1.34 (0.96, 1.87), p = 0.081
Small food stores	1200	Tertile 1	1.00	1.00	1.00
		Tertile 2	1.40 (1.09, 1.8), p = 0.009	1.18 (0.91, 1.54), p = 0.206	0.98 (0.72, 1.34), p = 0.922
		Tertile 3	1.96 (1.49, 2.58), p = 0.000	1.96 (1.48, 2.58), p = 0.000	1.51 (1.09, 2.11), p = 0.015

^1^Models adjusted for age, sex, country of birth, dominant household occupation, education, disability, area SEP

^2^Reference group for walking outcome: walking less than four times a week

^3^Reference group for physical activity outcome: insufficient level of activity

More specifically, there were significant positive associations observed between each destination type and walking frequency for: all destination types at 1200 m; cafes/takeaway stores, transport stops, supermarkets, community resources, educational destinations and small food stores at 800 m; café/takeaway food stores, sport facilities and transport stops at 400 m.

Associations were similar for physical activity sufficiency, with significant positive associations noted for: all destinations at 1200 m; educational destinations, café/takeaway stores, supermarkets, community resources, small food stores at 800 m; cafes/takeaway stores and transport stops at 400 m.

For both outcomes, strongest effects were observed for community resources at 800 m and 1200 m, and small food stores at 800 m and 1200 m.

Associations between destinations and sufficient physical activity were largely attenuated when walking was included in the models.

### Multi-level logistic regression analysis: Destination mix

For each buffer distance, the effect of destination mix on walking was significant (see Table 5). The odds of walking ≥ 4/week significantly increased for each additional destination type above the median for that type, at each buffer distance. At 400 m the odds increased by almost 10 % (OR 1.09, 95 % CI 1.03–1.16). The odds increased by 12 % for both 800 m (OR 1.12, 95 % CI 1.06–1.17) and 1200 m, (OR 1.12, 95 % CI 1.07–1.17). Effects of physical activity sufficiency were similar to those of walking, with the odds of being sufficiently physically active increasing significantly for each destination type above the median for that type. These effects were significant at both 800 m (OR 1.10, 95 % CI, 1.04–1.15) and 1200 m (OR 1.10, 95 % CI, 1.05–1.16).

**Table 5:** Multilevel logistic regression odds ratios for destination mix, at three distances^1^

Exposure variable	Buffer distance	Odds ratios (CI)		
		Walking^2^	Physical activity^3^	Physical activity adjusted for walking
Mix	400	1.09 (1.03, 1.16), p = 0.004	1.06 (0.99, 1.13), p = 0.090	1.02 (0.95, 1.10), p = 0.521
Mix	800	1.12 (1.06, 1.17), p = 0.000	1.10 (1.04, 1.15), p = 0.000	1.05 (0.99, 1.11), p = 0.141
Mix	1200	1.12 (1.07, 1.17), p = 0.000	1.10 (1.05, 1.16), p = 0.000	1.06 (1.00, 1.12), p = 0.044

^1^Models adjusted for age, sex, country of birth, dominant household occupation, education, disability, area SEP

^2^Reference group for walking outcome: walking less than four times a week

^3^Reference group for physical activity outcome: insufficient level of activity

Inclusion of walking in physical activity models led to attenuation of the association between destination mix and physical activity.

## Discussion

This analysis provides evidence that the presence of several different types of destinations in local neighbourhoods is associated with a greater likelihood of walking at least four times a week, and being sufficiently active. Community resources and small food stores showed the strongest associations with walking and physical activity: significant effects being observed at both the 800 m and 1200 m buffer, for both tertile 2 and tertile 3 (relative to tertile 1). For both outcomes, more moderate associations were observed for cafes/takeaway stores, supermarkets and educational destinations at most buffers.

Walking frequency largely attenuated the associations between the different destinations and sufficient physical activity, suggesting that increasing the number of destinations in areas has the potential to increase physical activity, largely through walking, such that more residents are sufficiently active for health.

There was also evidence that greater destination mix was associated with higher odds of walking at least four times a week: for each buffer distance, each additional destination type above the median was associated with a significant increase in the likelihood of walking at least once a week.

The strong associations between walking and community resources and small food stores within 1200 m of home suggest that people may walk up to 1200 m, (15 min) to access some services and destinations. The results for supermarkets and small food stores may suggest that having somewhere to buy basic food and household provisions within walking distance of home is important for walking.

Significant associations were observed for walking at all buffers for cafes/takeaway stores, and transport stops/stations – the most common destinations. The observed associations for public transport may be associated with the fact that people commonly access transport stops by walking. The comparative abundance of transport stops and café/takeaway stores may also mean that they are of sufficient quantity to enable the detection of an effect at 400 m.

Having a mixed set of destinations may offer the opportunity to achieve a range of shopping and other tasks in a single trip, thereby providing incentive to walk.

The importance of community resources in this study, is consistent with other research concluding that destinations offering and supporting social interaction were more predictive of walking than other destinations [19]. The findings in relation to the importance of food stores and schools/educational facilities are also consistent with the findings of other studies [16–18, 20, 21, 33].

In terms of distance effects, our results are similar to other studies. In Australia, local destinations within a 10 min walk from home have been found to be associated with walking for transport in adults [48]. Elsewhere, a Canadian study found that very few walks exceeded 1200 m [33].

### Strengths and limitations of this research

The use of three network buffers specific to individual respondents is a key strength of this analysis, as most previous studies have only examined one or two catchments/buffers. Information about distance is critical if we are to create new developments, or amend existing suburbs to make them more amenable to physical activity. Secondly, the destinations included in this analysis represent considerable breadth of possible destinations in local neighbourhoods. Thirdly, we have advanced the measurement of destination mix by accounting for variations in the distribution of different destinations.

There are some limitations of the data. The cross-sectional nature of this study means that causality cannot be inferred from associations. Secondly, the determinants of walking can vary according to walking purpose. While walking to a destination can be classified as walking for transport, the outcome measure used was total walking frequency in the past month (we did not distinguish between recreational and transport walking). This risks error in estimates of the effect of the exposure variables – most likely a bias toward null. Sensitivity analysis conducted by removing those who only reported walking for recreation however, resulted in stronger effects for destinations. Importantly, it is often difficult for both respondents (and analysts) to distinguish between walking trips on the basis of purpose. For example, it is difficult to assign walking purpose to a person walking to a café or library.

The walking outcome measure was based on walking for 10 min or more (respondents were asked how often in the last month they had walked for 10 min or more). For most people, this equates to a distance of approximately 800 m. Consequently, walking trips to destinations within 400 m may not have been captured, and the effect of destinations within 400 m may be underestimated. However it is possible that some walking within 400 m was captured through trip chaining (walking to, and between multiple destinations), and making round trips (rather than one-way journeys), as the walking carried out by a person walking to several destinations within 400 m may amount to 10 min or more. Certainly this notion of trip-chaining is supported by the significance of results for destination mix within 400 m of home. Also in relation to the walking outcome, we have no measure of the intensity of respondents’ walking and therefore do not know if people walked at sufficient intensity to benefit their health.

There is also a risk of confounding due to residential self-selection. Self-selection may occur when people who perceive that there are benefits to physical activity and walking choose to live in (self-select into) neighbourhoods that support such activities [32, 49]. However we controlled for many potential demographic and socio-economic confounders that predict attitudes, perceptions and behaviours in relation to both physical activity and walking.

Finally, we did not have information about where people walked or were physically active. Given that the majority of the sample was in the labour force, many people may have walked close to work. We did not have information about destinations close to the workplace and therefore we do not know the extent to which they exert an influence over walking.

### Implications for policy and practice

These results have important implications for policy and practice. The results provide guidance for urban planners, suggesting that several different types of destinations may encourage walking. The associations between physical activity and destinations suggest that destinations may encourage physical activity to a level sufficient to confer health and lifestyle benefits to residents. Given that cycling, another mode of active transport that might be used to reach destinations, is very infrequent among Australian adults [50], it is likely that much of the physical activity measured here is derived through walking.

The different associations between destination types and physical activity and walking provide a guide for priorities in the planning of shops and services. In terms of a hierarchy of importance, the results suggest that small food stores and destinations offering opportunity for social interaction such as community resources may induce greatest influence on walking and physical activity. Evidence from this same study found the presence of healthy food stores close to home is associated with healthy eating [41]. This suggests a two-pronged benefit of having supermarkets or small shops within walking distance of home: with benefits in terms of physical activity, and healthy eating.

The strength and significance of the associations between the outcomes and shop and service destinations at 800 m and 1200 m is important in guiding decisions about proximity. The results suggest that destinations up 1200 m may still exert an influence on walking and physical activity (which from both an economic and public health point of view is advantageous). Additionally, when planning new suburbs, and working within existing suburbs it is important to include a mix of destination types, as a mix of destinations is associated with an increase in the odds of walking and physical activity.

While these results have implications for planning practices, it is also important to acknowledge that the location of destinations is driven by many factors including (but not limited to) population density, street connectivity, and pedestrian safety. Increasing walkable destinations alone is unlikely to promote walking and physical activity if these other aspects of pedestrian infrastructure are unsupportive.

## Conclusion

These results build on previous studies and add to the weight of evidence regarding the importance of destinations for walking and physical activity. In particular, key destinations such as small food stores, community resources and schools within 800 m-1200 m of home may offer the greatest scope to promote local physical activity and walking. The results also suggest that greatest walking and physical activity potential is realized if destinations are mixed.

### Ethics approval

The VicLANES project design was approved by the La Trobe University Human Ethics Committee (#02-130). Participants received an information pack, along with their survey in the mail. This advised them: of the risks and benefits of participating; that their participation was voluntary; of the ways that the data would be used; of the strict procedures to protect confidentiality and ensure anonymity. Return of completed surveys was considered indicative of consent - a procedure approved by the Latrobe University Human Ethics Committee.

## Additional files

Additional file 1: Table S1. Types and sources of destination data. (DOC 46 kb) Additional file 2: Table S2. Summary of destination exposures and covariates. (DOC 39 kb)

## Footnotes

1 TAFE stands for ‘technical and further education’. In Australia, TAFE institutions provide vocational training and courses in fields such as engineering, business, finance, hospitality, and tourism.

## References

[1] Centers for Disease Control and Prevention. Physical activity and health: a report for the Surgeon General. Atlanta: US Department of Health and Human Services; 1996.

[2] Lagerros YT (2009). Physical activity—the more we measure, the more we know how to measure. Eur J Epidemiol.

[3] Australian Bureau of Statistics. Physical Activity in Australia: A Snapshot, 2004–05 (Catalogue no. 4835.0.55.001)**.** Canberra: ABS; 2006.

[4] Armstrong T, Bauman A, Davies J (2000). Physical activity patterns of Australian adults. Results of the 1999 National Physical Activity Survey.

[5] Australian Bureau of Statistics. National Health Survey: Summary of Results, 2007–2008 (Catalogue no. 4364.0)**.** Canberra: Australian Bureau of Statistics; 2011.

[6] Siegel PZ, Brackbill RM, Heath GW (1995). The epidemiology of walking for exercise: implications for promoting activity among sedentary groups. Am J Public Health.

[7] Simpson ME, Serdula M, Galuska DA, Gillespie C, Donehoo R, Macera C, Mack K (2003). Walking trends among U.S. adults: The Behavioral Risk Factor Surveillance System, 1987–2000. Am J Prev Med.

[8] Zheng H, Orsini N, Amin J, Wolk A, Nguyen VTT, Ehrlich F (2009). Quantifying the dose–response of walking in reducing coronary heart disease risk: meta-analysis. Eur J Epidemiol.

[9] Hamer M, Chida Y (2008). Walking and primary prevention: a meta-analysis of prospective cohort studies. Br J Sports Med.

[10] Murphy MH, Nevill AM, Murtagh EM, Holder RL (2007). The effect of walking on fitness, fatness and resting blood pressure: a meta-analysis of randomised, controlled trials. Prev Med.

[11] Ewing R, Cervero R (2010). Travel and the built environment. J Am Plann Assoc.

[12] Duncan M, Winkler E, Sugiyama T, Cerin E, duToit L, Leslie E, Owen N (2010). Relationships of land use mix with walking for transport: Do land uses and geographical scale matter?. J Urban Health.

[13] Turrell G, Haynes M, Wilson L-A, Giles-Corti B (2013). Can the built environment reduce health inequalities? A study of neighbourhood socioeconomic disadvantage and walking for transport. Health Place.

[14] Owen N, Cerin E, Leslie E, duToit L, Coffee N, Frank LD, Bauman AE, Hugo G, Saelens BE, Sallis JF (2007). Neighborhood walkability and the walking behavior of Australian adults. Am J Prev Med.

[15] Frank LD, Engelke PO, Schmid TL (2003). Health and community design: the impact of the built environment on physical activity.

[16] Moudon AV, Lee C, Cheadle AD, Garvin C, Johnson D, Schmid TL, Weathers RD, Lin L (2006). Operational definitions of walkable neighborhood: theoretical and empirical insights. J Phys Act Health.

[17] Lin L, Moudon AV (2010). Objective versus subjective measures of the built environment, which are most effective in capturing associations with walking?. Health Place.

[18] McCormack GR, Giles-Corti B, Bulsara M (2008). The relationship between destination proximity, destination mix and physical activity behaviors. Prev Med.

[19] Nathan A, Pereira G, Foster S, Hooper P, Saarloos D, Giles-Corti B (2012). Access to commercial destinations within the neighbourhood and walking among Australian older adults. Int J Behav Nutr Phys Act.

[20] Tilt JH, Unfried TM, Roca B (2007). Using objective and subjective measures of neighborhood greenness and accessible destinations for understanding walking trips and BMI in Seattle, Washington. Am J Health Promot.

[21] Cerin E, Leslie E, Toit L, Owen N, Frank LD (2007). Destinations that matter: associations with walking for transport. Health Place.

[22] Bentley R, Jolley D, Kavanagh A (2010). Local environments as determinants of walking in Melbourne, Australia. Soc Sci Med.

[23] De Bourdeauduij I, Sallis JF, Saelens BE (2003). Environmental correlates of physical activity in a sample of Belgian adults. Am J Health Promot.

[24] Inoue S, Murase N, Shimomitsu T, Ohya Y, Odagiri Y, Takamiya T, Ishii K, Katsumura T, Sallis JF (2009). Association of physical activity and neighborhood environment among Japanese Adults. Prev Med.

[25] Wang Z, Lee C (2010). Site and neighborhood environments for walking among older adults. Health Place.

[26] Krizek KJ, Johnson PJ (2006). Proximity to trails and retail: effects on urban cycling and walking. J Am Plann Assoc.

[27] King WC, Brach JS, Belle S, Killingsworth R, Fenton M, Kriska AM (2003). The relationship between convenience of destinations and walking levels in older women. Am J Health Promot.

[28] Michael YL, Green MK, Farquhar SA (2006). Neighborhood design and active aging. Health Place.

[29] Witten K, Blakely T, Bagheri N, Badland H, Ivory V, Pearce J, Mavoa S, Hinckson E, Schofield G (2012). Neighborhood built environment and transport and leisure physical activity: findings using objective exposure and outcome measures in New Zealand. Environ Health Perspect.

[30] Davis M, Fox K, Hillsdon M, Coulson J, Sharp D, Stathi A, Thompson J (2011). Getting out and about in older adults: the nature of daily trips and their association with objectively assessed physical activity. Int J Behav Nutr Phys Act.

[31] Suminski RR, Poston WSC, Petosa RL, Stevens E, Katzenmoyer LM (2005). Features of the neighborhood environment and walking by U.S. adults. Am J Prev Med.

[32] Handy S, Cao X, Mokhtarian P (2006). Self-selection in the relationship between the built environment and walking. J Am Plann Assoc.

[33] Millward H, Spinney J, Scott D (2013). Active-transport walking behavior: destinations, durations, distances. J Transport Geogr.

[34] McCormack GR, Friedenreich C, Sandalack BA, Giles-Corti B, Doyle-Baker PK, Shiell A (2012). The relationship between cluster-analysis derived walkability and local recreational and transportation walking among Canadian adults. Health Place.

[35] Giles-Corti B, Bull F, Knuiman M, McCormack G, Van Niel K, Timperio A, Christian H, Foster S, Divitini M, Middleton N, Boruff B (2013). The influence of urban design on neighbourhood walking following residential relocation: Longitudinal results from the RESIDE study. Soc Sci Med.

[36] Forsyth A, Hearst M, Oakes JM, Schmitz KH (2008). Design and Destinations: factors influencing walking and total physical activity. Urban Studies.

[37] McConville ME, Rodríguez DA, Clifton K, Cho G, Fleischhacker S (2011). Disaggregate land uses and walking. Am J Prev Med.

[38] Sugiyama T, Neuhaus M, Cole R, Giles-Corti B, Owen N (2012). Destination and route attributes associated with adults' walking: a review. Med Sci Sports Exerc.

[39] King T, Kavanagh A, Jolley D, Turrell G, Crawford D (2005). Weight and place: a multilevel cross-sectional survey of area-level social disadvantage and overweight/obesity in Australia. Int J Obes (Lond).

[40] Kavanagh AM, Goller JL, King TL, Jolley D, Crawford D, Turrell G (2005). Urban area disadvantage and physical activity: a multilevel study in Melbourne, Australia. J Epidemiol Community Health.

[41] Mason KE, Bentley RJ, Kavanagh AM (2013). Fruit and vegetable purchasing and the relative density of healthy and unhealthy food stores: evidence from an Australian multilevel study. J Epidemiol Community Health.

[42] King T, Thornton L, Bentley R, Kavanagh A (2012). Does parkland influence walking? The relationship between area of parkland and walking trips in Melbourne, Australia. Int J Behav Nutr Phys Act.

[43] Australian Electoral Commission (2005). Measuring the accuracy of the electoral rolls and testing the effectiveness of continuous roll update: a report on the fieldwork from February-March 2004.

[44] Australian Institute of Health and Welfare (2003). The active Australia survey: a guide and manual for implementation, analysis and reporting.

[45] Department of Health (2004). At least five a week: evidence on the impact of physical activity and its relationship to health.

[46] Australian Bureau of Statistics. Australian Health Survey: Physical Activity, 2011–12 (Catalogue No. 4364.0.55.004). Canberra: Australian Bureau of Statistics; 2013.

[47] Bauman A, Ford I, Armstrong T (2001). Trends in population levels of reported physical activity in Australia, 1997, 1999 and 2000.

[48] Sugiyama T, Inoue S, Cerin E, Shimomitsu T, Owen N. Walkable area within which destinations matter: differences between Australian and Japanese cities. Asia Pac J Public Health. 2012. 10.1177/1010539512466911 23188878

[49] Cao X, Mokhtarian PL, Handy S. Examining the impacts of residential self-selection on travel behaviour: a focus on empirical findings. Transport Reviews. 2009;29:359–95.

[50] Australian Bureau of Statistics. Australian Social Trends, July 2013 (Catalogue no. 4102.0). Canberra: Australian Bureau of Statistics; 2014.

