# Supplementary material for: Does the presence and mix of destinations influence walking and physical activity?
Source: Int J Behav Nutr Phys Act. 2015 Sep 17;12:115. doi: 10.1186/s12966-015-0279-0 (PMC4573483; doi:10.1186/s12966-015-0279-0)
Supplement: Additional file 1: Table S1. — Types and sources of destination data. (DOC 46 kb) [file 12966_2015_279_MOESM1_ESM.doc]

**Additional file 1: Table S1**: Types and sources of destination data

| **Data Provider** | **Data** | **Destination category** |
| --- | --- | --- |
| Street directories (Ausway Pty Ltd) | - Childcare centres & kindergartens | Education |
|  | - Other places of education |  |
|  | - Schools |  |
|  | - Public libraries | Community resources |
|  | - Galleries, cinemas, theatres, museums |  |
|  | - Neighbourhood houses |  |
|  | - Community health centres |  |
|  | - Community centres |  |
|  | - Places of worship/churches |  |
|  | - Maternal and child health centres |  |
|  | - Post offices |  |
|  | - Tennis courts | Sport |
|  | - Swimming pools |  |
| VicLANES environmental audits | - Supermarkets | Supermarkets |
|  | - Fruit & vegetable stores - Butchers - Bakeries - Ethnic food stores - Specialty food stores - Minor/convenience food stores - Other food stores | Small food stores |
|  | - Café/takeaway restaurants | Café/takeaway restaurants |
| Metlink (Public transport operator for the Victorian State Government) | - Tram stops - Bus stops | Transport |
| PSMA (Public Service Mapping Agency) Australia Limited | - Railway lines - Railway stops - Tram/light rail lines |  |
