# Supplementary material for: Does the presence and mix of destinations influence walking and physical activity?
Source: Int J Behav Nutr Phys Act. 2015 Sep 17;12:115. doi: 10.1186/s12966-015-0279-0 (PMC4573483; doi:10.1186/s12966-015-0279-0)
Supplement: Additional file 2: Table S2. — Summary of destination exposures and covariates. (DOC 39 kb) [file 12966_2015_279_MOESM2_ESM.doc]

**Additional file 2: Table S2: Summary of destination exposures and covariates[[1]](#footnote-2)**

|  | **400m buffer** | **800m buffer** | **1200m buffer** |
| --- | --- | --- | --- |
| **Educational facilities** | +single adults/no children  +mid area disadvantage | +aged 35-44 yrs  +bachelor degree or higher  +DomOcc[[2]](#footnote-3)=professional  +single adult/no children  +has injury/disability  -55-64 yrs  -not born in Aust.  -low area disadvantage | +35-44 years  +born in Aust.  +bachelor degree+  +DomOcc=professional  +single adults/no children |
| **Café/takeaway stores** | +single adults/no children  +injury/disability  +high area disadvantage  -two or more adults with children | +35-44 years & +65 years +  +born in Australia  +bachelor degree+  +DomOcc=professional  +single adults/no children  +high area disadvantage  -two adults with children | +24-34 years & +65years +  +Bachelor degree +  +DomOcc=professional  +single adults/no children  +injury or disability  +high/mid area disadvantage  -two adults with children |
| **Transport stops/stations**  (train stations, bus and tram stops) | +single adults/no children  +high area disadvantage | +35-44 years & +65years+  +DomOcc=Not in labour force  +single adults/no children  +injury/disability  +high area disadvantage | +35-44 years  +born in Australia  +single adults/no children |
| **Supermarkets** | +65years+  +DomOcc=not in labour force  +single adults/no children  +high and mid-level disadvantage | +65 years+  +single adults/no children  +High and mid level disadvantage-35-44 years  - two or more adults with/without children  -low area disadvantage | +single adults/no children  +High and mid level disadvantage  -45-54 years  -two or more adults and children  -low area disadvantage |
| **Sporting facilities**  (tennis courts, swimming pools) | +65 years +  +diploma  +bachelor degree or higher  +single adults/no children  +injury/disability  +high area disadvantage | +65 years +  +DomOcc=not in labour force  +single adults/no children  +high area disadvantage | +65 years +  +diploma  +bachelor degree or higher  +single adults/no children  +injury/disability  +high area disadvantage  +DomOcc=not in labour force or professional |
| **Community resources** (libraries, neighbourhood houses, places of worship, galleries, museums, theatres, maternal and child health centres, post offices, community health centres, community centres) | +35-44 years &+65 years +  +born in Australia  +single adults/no children  +injury/disability  +high area disadvantage  - two or more adults with/without children  -low area disadvantage | +35-44 years  +born in Australia  +bachelor degree+  +DomOcc=professional  +single adults/no children  +high area disadvantage  -45-54 years & -55-64 years  -no post school qualifications  -two or more adults with children  -low area disadvantage | +bachelor degree+  +DomOcc=professional  +single adults/no children  +High and mid level disadvantage  -no post school qualifications  -vocational qualifications -two or more adults with children  -low area disadvantage |
| **Small food stores** (greengrocers, convenience stores, bakeries, butchers, small grocery stores, speciality food stores) | +DomOcc=not in labour force  +single adults/no children  +injury/disability  +high area disadvantage  -DomOcc=professional  -low area disadvantage | +35-44 years & +65 years +  +bachelor degree+  +DomOcc=professional  +single adults/no children  +high area disadvantage  -55-64 years  --two or more adults with/without children  -low area disadvantage | +25-34 years  +bachelor degree+  +DomOcc=professional  +single adults/no children  +mid and high area disadvantage  -18-24 years  -not born in Australia  -DomOcc=white collar  -two or more adults with children  -no injury/disability  -low area disadvantage |

1. ‘+’ denotes high exposure (high representation in the high exposure group), while ‘-‘ denotes low exposure (high representation in the low exposure group) [↑](#footnote-ref-2)
2. DomOcc=Dominant household occupation [↑](#footnote-ref-3)
